# Impact of Frailty on Medical and Long-Term Care Expenditures for the Elderly Age 75 or Over in Japan

**DOI:** 10.1093/geroni/igaa057.569

**Published:** 2020-12-16

**Authors:** Hiroto Yoshida, Yuriko Kihara

**Affiliations:** 1 Tohoku Bunka Gakuen University, Sendai, Japan; 2 Japan Health Care College, Eniwa, Hokkaido, Japan

## Abstract

This study examined the impact of frailty on medical and long-term care expenditures in an older Japanese population. The subjects were those aged 75 years and over who responded to the survey (March 2018) in Bibai, Hokkaido, Japan (n=1,203) and have never received certification of long-term care insurance at the survey. We followed up 867 individuals (72.1%) until the end of December 2018 (10 month-period). We defined frailty as a state in performing 4 items and over of 15 items which were composed of un-intentional weight loss, history of falls, etc. Among 867 subjects, 233 subjects (26.9%) were judged to be frailty group, and 634 subjects (73.1%) non-frailty group. We compared period to the new certification of long-term care insurance (LTCI), accumulated medical and long-term care expenditures adjusted for age and gender between the two groups during the follow-up period. Cox proportional hazard models were used to examine the association between baseline frailty and the new certification of LTCI. The relative hazard ratio (HR) was higher in frailty group than non-frailty group (HR=3.51, 95% CI：1.30-9.45, P=.013). The adjusted mean accumulated medical and long-term care expenditures per capita during the follow-up were significantly (P=.002) larger for those in the frailty group (629,699 yen), while those in the non-frailty group were 450,995 yen. We confirmed strong economic impact of frailty in the elderly aged 75 or over in Japan.

